# Trajectory of Spike-Specific B Cells Elicited by Two Doses of BNT162b2 mRNA Vaccine

**DOI:** 10.3390/cells12131706

**Published:** 2023-06-23

**Authors:** Annalisa Ciabattini, Gabiria Pastore, Simone Lucchesi, Giorgio Montesi, Simone Costagli, Jacopo Polvere, Fabio Fiorino, Elena Pettini, Arianna Lippi, Leonardo Ancillotti, Mario Tumbarello, Massimiliano Fabbiani, Francesca Montagnani, Donata Medaglini

**Affiliations:** 1 Laboratory of Molecular Microbiology and Biotechnology, Department of Medical Biotechnologies, University of Siena, 53100 Siena, Italy; gabiria.pastore@unisi.it (G.P.); lucchesi5@student.unisi.it (S.L.); giorgio.montesi@unisi.it (G.M.); simone.costagli@unisi.it (S.C.); jacopo.polvere@student.unisi.it (J.P.); fiorino4@unisi.it (F.F.); pettini5@unisi.it (E.P.); donata.medaglini@unisi.it (D.M.); 2 Department of Medicine and Surgery, LUM University “Giuseppe Degennaro”, 70010 Casamassima, Italy; 3 Infectious and Tropical Diseases Unit, Department of Medical Sciences, University Hospital of Siena, 53100 Siena, Italy; arianna.lippi@unifi.it (A.L.); leonardo.ancillotti@unifi.it (L.A.); mario.tumbarello@unisi.it (M.T.); m.fabbiani@ao-siena.toscana.it (M.F.); francesca.montagnani@unisi.it (F.M.); 4 Department of Medical Biotechnologies, University of Siena, 53100 Siena, Italy

**Keywords:** B cell response, mRNA vaccination, computational analysis, SARS-CoV-2, COVID-19

## Abstract

The mRNA vaccines for SARS-CoV-2 have demonstrated efficacy and immunogenicity in the real-world setting. However, most of the research on vaccine immunogenicity has been centered on characterizing the antibody response, with limited exploration into the persistence of spike-specific memory B cells. Here we monitored the durability of the memory B cell response up to 9 months post-vaccination, and characterized the trajectory of spike-specific B cell phenotypes in healthy individuals who received two doses of the BNT162b2 vaccine. To profile the spike-specific B cell response, we applied the tSNE and Cytotree automated approaches. Spike-specific IgA^+^ and IgG^+^ plasmablasts and IgA^+^ activated cells were observed 7 days after the second dose and disappeared 3 months later, while subsets of spike-specific IgG^+^ resting memory B cells became predominant 9 months after vaccination, and they were capable of differentiating into spike-specific IgG secreting cells when restimulated in vitro. Other subsets of spike-specific B cells, such as IgM^+^ or unswitched IgM^+^IgD^+^ or IgG^+^ double negative/atypical cells, were also elicited by the BNT162b2 vaccine and persisted up to month 9. The analysis of circulating spike-specific IgG, IgA, and IgM was in line with the plasmablasts observed. The longitudinal analysis of the antigen-specific B cell response elicited by mRNA-based vaccines provides valuable insights into our understanding of the immunogenicity of this novel vaccine platform destined for future widespread use, and it can help in guiding future decisions and vaccination schedules.

## 1. Introduction

The COVID-19 pandemic has affected millions of people worldwide, causing significant morbidity and mortality (https://covid19.who.int/). Vaccination has emerged as a crucial strategy in restraining the severity of the disease, and several vaccines have been developed to combat the COVID-19 virus. These vaccines, employing different mechanisms of action, have shown varying levels of efficacy and safety [1]. Among these, RNA technology represents a revolution in vaccine production as it enables faster and less expensive production compared to traditional methods and can be easily adapted to address virus mutations. RNA vaccines are expected to remain a critical approach in the fight against infectious diseases, not only for COVID-19, but also for other pathogens such as influenza and HIV, as well as for non-infectious diseases such as cancer and autoimmune disorders [2,3,4,5]. However, further research is still needed to fully evaluate the immunogenicity, safety, and efficacy of this technology. A critical question surrounding COVID-19 vaccination with mRNA-based vaccines is the duration of the immune response elicited. Current evidence suggests that vaccinated individuals maintain robust protection against severe illness and mortality for a minimum of 6 months [6]. However, the effectiveness of the vaccines in preventing infection and mild symptoms may diminish over time [7]. Consequently, public health agencies have recommended the administration of booster doses starting at 4–6 months after completing the primary vaccination series to enhance protection against severe illness and death caused by COVID-19. Older and vulnerable populations have been prioritized for booster immunization due to their pathologies or immunosuppressive treatments that compromise immune responsiveness [8,9,10,11,12,13]. Numerous studies have demonstrated the critical role of the third vaccine dose for vulnerable individuals, such as those with myelofibrosis, undergoing hemodialysis, or recipients of hematopoietic cell transplants, who exhibited a weaker or slower immune response to the initial vaccination cycle [11,14,15,16,17]. Despite strong recommendations for the third dose only 30% of the global population has received the booster dose, while 65.5% of people have completed the primary vaccination cycle with two doses (https://covid19.who.int/table; accessed on 1 May 2023). Therefore, it is crucial to investigate the persistence of immune memory following the initial vaccination schedule. As mRNA-based platforms are being used for the first time, various aspects regarding the safety, mechanisms of action of the nanoparticles [18], and the antibody response have been extensively examined from the outset in both healthy and fragile subjects [13,19,20]. However, other aspects, such as the long-term persistence of immune memory [21,22,23,24] and the hybrid immunity induced by concurrent viral infection [25,26], remain under investigation and necessitate continuous updates. 

Immune memory is the immunological mechanism that protects individuals against reinfection. It is the primary target of vaccination, as memory B cells (MBCs) can rapidly reengage upon re-encountering the antigen, differentiating into antibody-secreting cells capable of combating microbial infections [27]. Long-lived plasma cells, originating from the germinal center and residing in the bone marrow, are also integral components of the memory cell pool [28]. These cells exhibit higher antibody avidity and secretion rates compared to their short-lived counterparts generated primarily through extrafollicular reactions. Vaccination also induces memory T cells, as observed with numerous COVID-19 vaccines [29,30,31]. Reactivated memory T cells are able to kill infected cells, thus preventing viral multiplication and spread. SARS-CoV-2 infection and/or vaccination studies have revealed the persistence of memory cells in unvaccinated infected patients [32,33,34] and vaccinated subjects [21,23,24,31,35] when antibody levels naturally decline over time. In a previous work, we demonstrated the generation and persistence of peripheral spike-specific MBC and circulating antibodies up to 6 months after the first cycle of vaccination with the BNT162b2 vaccine in a cohort of SARS-CoV-2-naïve healthy subjects [35]. Furthermore, the long-term persistence of germinal center reaction into axillary draining lymph nodes, together with the generation of high affinity-MBCs and long-lived plasma cells, has been demonstrated in humans who received the two-dose series of BNT162b2 vaccination [36]. 

Here, we characterize the dynamics and magnitude of the spike-specific B cell response over a 9-month period post-vaccination, in a cohort of healthy subjects following the administration of the second dose of the BNT162b2 mRNA vaccine. Notably, this cohort was selected based on the absence of nucleocapsid-specific antibodies at all analyzed time points, making it an ideal population for profiling the antigen-specific B cell response specifically induced by the novel mRNA-based vaccination platform, independent of any confounding effects of hybrid immunity resulting from natural infection. 

## 2. Materials and Methods

### 2.1. Study Design

Plasma and peripheral blood mononuclear cells (PBMCs) samples were obtained from a total of 30 healthcare workers (HCWs) aged 26–63 years who received two doses of the BNT162b2 (Pfizer-BioNTech; Comirnaty) vaccine, 3 weeks apart. Exclusion criteria included pregnancy, previously documented SARS-CoV-2 infection, and immunocompromising comorbidities (congenital, acquired, or drug-related). All participants provided written informed consent before participation in the study. Study participants were recruited at the Infectious and Tropical Diseases Unit, Azienda Ospedaliera Universitaria Senese (Siena, Italy). The study was performed in compliance with all relevant ethical regulations, and the protocol was approved by the local Ethical Committee for Clinical Experimentation of Regione Toscana Area Vasta Sud-Est (CEAVSE), protocol code 18869 IMMUNO_COV v1.0 of 18 November 2020, approved on 21 December 2020. Clinical data collection and management were carried out using the software REDCap (Research Electronic Data Capture, Vanderbilt University; Nashville, TN, USA). 

### 2.2. PBMCs Isolation

Venous blood samples were collected in heparin-coated BD Vacutainer blood tubes (Becton Dickinson, Franklin Lakes, NJ, USA. PBMCs were isolated using density-gradient sedimentation using Ficoll-Paque (Lymphoprep, Meda, Italy). Cells were gently resuspended with warm cell recovery medium [10% DMSO (Thermo Fisher Scientific; Waltham, MA, USA) and 90% heat-inactivated fetal bovine serum (Sigma Aldrich; St. Louis, MO, USA) and then rapidly transferred to cryovials that were incubated o.n. at −80 °C using an isopropanol freezing container. Vials were cryopreserved in liquid nitrogen. Plasma samples were stored at −80 °C. Serological and flow cytometry analyses were performed in frozen/thawed samples.

### 2.3. Multiparametric Flow Cytometry

SARS-CoV-2-specific B cells were identified and characterized in thawed PBMC with flow cytometry. Biotinylated recombinant wild-type SARS-CoV-2 Spike S1 + S2 ECD (Sino Biological, Beijing, China; sequence YP_009724390.1, Val 16-Pro1213), and RBD domain (BioLegend; San Diego, CA, USA) were tetramerized with streptavidin (SA) fluorescently labeled with R-Phycoerythrin (PE; Thermofisher) or Allophycocyanin (APC; Thermofisher, Waltham, MA, USA), as previously described [37]. Briefly, 2 million PBMCs were blocked with BD human FC block (BD Biosciences; Becton Dickinson, Franklin Lakes, NJ, USA) and stained for 30 min at 4 °C with the following antibody–fluorochrome panel: CD3-BV650 (clone SK7), CD19-BUV395 (clone SJ25C1), IgM-BV605 (clone G20-127), IgD-BV711 (clone IA6-2), CD20-APC-H7 (clone 2H7), CD27-BV786 (clone M-T271), CD21-FITC (clone B-ly4), CD38-BUV737 (clone HB7), IgG-PE-Cy7 (clone G18-145, all from Becton Dickinson, Franklin Lakes, NJ, USA), and IgA-VioBlue (clone IS11-8E10, Miltenyi Biotec; Bergisch Gladbach, Germany). After staining, cells were labeled with Zombie Aqua™ Fixable Viability Kit (Thermofisher) according to the manufacturer’s instructions and fixed with BD fixation solution (BD Biosciences). All antibodies were titrated for optimal dilution. About 1 × 10^6^ cells were acquired for each sample with an SO LSRFortessa X20 flow cytometer (BD Biosciences). Manual data analysis was performed using FlowJo v10.8.1 (TreeStar, Ashland, OR, USA).

### 2.4. t-SNE Analysis

The B cell population analyzed was gated as live, singlet, CD3^−^/CD19^+/low^ cells using FlowJo v10 (TreeStar, USA). For each sample, an equal amount of CD3^−^/CD19^+/low^ cells (*n* = 5000) was randomly sampled, starting from an equal number of samples (*n* = 12) for each time point. Files were exported from FlowJo as uncompensated flow cytometry standard (FCS) files. FCS files were imported into R environment (v4.1.3, R Core Team, Vienna, Austria) as flowSet objects, which were then compensated with FlowCore package 2.6.0 [38] and transformed using *logicleTransform* function [39]. A t-Distributed Stochastic Neighbor Embedding (t-SNE) [40] dimensionality reduction was performed with Rtsne package v0.15 [40]. Expression values of each marker were normalized as z-scores (mean = 0 and standard deviation = 1). Then, the t-SNE algorithm was run, setting perplexity = 100, selected as the optimal parameter value in a range between 5 and 200. B cells were also manually analyzed with FlowJo, and labels of different B cell populations were imported into R environment using the GetFlowJoLabels function from the FlowSOM package (v2.2.0) [41]. Contour plots of individual B cell populations were displayed with the function “Contour” from the FlowViz package (v1.58.0). 

### 2.5. Trajectory Analysis

Spike^+^ RBD^+^ cells were gated within CD3^−^/CD19^+/low^ cells using FlowJo v10 (TreeStar, USA) and imported into R environment as compensated FCS files, for a total of 4700 cells. Spike^+^ RBD^+^ cells per sample ranged between 41 and 324 at d28, 11 and 228 at M3, and 25 and 249 at M9. Quality control, normalization, and sample merging were all performed using the CytoTree package (v1.0.3) [42]. After correcting for batch effect using the sva package (v3.46.0) [43], unsupervised clustering was performed using the FlowSom package (v2.2.0), and 36 clusters and 9 meta clusters were set up. The t-SNE dimensionality reduction was applied to both cells and clusters to construct trajectories for all clusters using a Minimum Spanning Tree approach implemented in CytoTree. The analysis of differentially expressed markers, including CD27, CD21, CD20, CD19, CD38, IgA, IgD, IgM, and IgG, performed using both CytoTree and flowDensity (v1.32.0) [44], allowed the identification of the different cellular phenotypes present within the Spike^+^ RBD^+^ population.

### 2.6. B Cell ELISpot

PBMCs, collected from subjects 9 months following vaccination, were evaluated for IgG production using the Human IgG Single-Color Enzymatic ELISpot assay (CTL Europe GmbH, Bonn, Germany). The protocol was performed according to the manufacturer’s instructions. Briefly, 2 × 10^6^ PBMCs/mL were stimulated with a polyclonal B cell Stimulator for 4 days, and then cells were transferred onto multiscreen filter 96-well plates, coated with recombinant wild-type SARS-CoV-2 Spike S1 + S2 (Sino Biological, 10 μg/mL) or anti-Ig capture antibody or an unrelated antigen and incubated o.n. Plates were then incubated with anti-human IgG detection solution and with Tertiary Solution and developed by adding Blue Developer Solutions. The number of spots was determined by plate scanning and analyzed with an Immunospot S6 Ultimate Analyzer (CTL Europe GmbH).

### 2.7. ELISA and ACE2/RBD Inhibition Assay

Maxisorp microtiter plates (Nunc, Roskilde, Denmark) were coated with recombinant wild-type SARS-CoV-2 Spike S1 + S2 ECD (Sino Biological), as previously described [14] or with the SARS-CoV-2 nucleoprotein (1 µg/mL, Sino Biological). Briefly, after blocking, plates were added with plasma samples for 1 h at RT. Anti-human horseradish peroxidase (HRP)-conjugated IgG (diluted 1:6000), IgM (diluted 1:2000), or IgA (diluted 1:4000; all from Southern Biotech) were added for 1 h, and plates were developed with 3,3′,5,5′-Tetramethylbenzidine (TMB; Thermo Fisher Scientific) substrate. Absorbance at 450 nm was measured on a Multiskan FC Microplate Photometer (Thermo Fisher Scientific). WHO international positive (NIBSC 20/150 and 20/144 for S and N, respectively) and negative (NIBSC 20/142) controls were added in duplicate to each plate as internal controls for assay reproducibility and to set the positive threshold.

ACE2/RBD inhibition was tested with a SARS-CoV-2 surrogate virus neutralization test (sVNT) kit (cPass™, GenScript Biotech, Rijswijk, The Netherlands), according to the manufacturer’s protocol, as already described [35]. Briefly, diluted plasma samples and positive and negative controls were mixed 1:1 with diluted HRP-RBD (either Wuhan, Delta, or Omicron BA.1 variants, Sino Biologicals) for 30 min at 37 °C, and then each mixture was added to ACE2-coated flat-bottom 96-well plates. TMB solution was added to each well, and plates were developed for 15 min at RT. The absorbance was measured at 450 nm on a Multiskan FC Microplate Photometer (Thermo Fisher Scientific). Results of the ACE2/RBD inhibition assay are expressed as percentage inhibition = (1—sample OD value/negative control OD value) × 100. Inhibition values ≥30% are regarded as positive results, as indicated by the manufacturer.

### 2.8. Statistics

The Kruskall–Wallis test, followed by Dunn’s post-test for multiple comparative tests, was used for assessing statistical between frequencies of S^+^RBD^+^ B cells and subsets at different time points. The Mann–Whitney test was used for assessing the statistical difference between spike-specific and unrelated antigen-specific B cells in ELISPOT data. A *p*-value ≤ 0.05 was considered significant. Analyses were performed using GraphPad Prism v9 (GraphPad Software, San Diego, CA, USA).

## 3. Results

### 3.1. Durability of Spike^-^Specific Memory B Cells over Time

The spike-specific B cell response in healthy subjects vaccinated with two doses of the BNT162b2 mRNA vaccine was analyzed starting from 7 days up to 9 months after the second dose administration. To exclude a booster effect elicited by a possible SARS-CoV-2 infection, all participants were assessed for anti-nucleoprotein antibodies at all time points, and only subjects with negative results were included in the present study (Supplementary Figure S1). 

**Figure 1:**
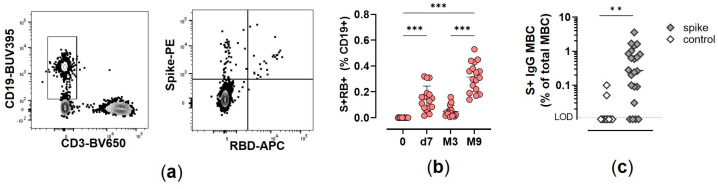
Spike-specific B cells in vaccinated subjects. (**a**) Flow cytometry analysis of S^+^RBD^+^ cells gated on live CD19^+/low^ cells, and (**b**) their frequency at baseline (0), 7 days (d7), 3 (M3), and 9 (M9) months after the second vaccine dose; Kruskall–Wallis test, followed by Dunn’s post-test for multiple comparative tests, was used for assessing statistical differences between cell frequency at different time points. *** *p* ≤ 0.001. (**c**) Spike-specific IgG-producing cells, assessed by B cell ELISPOT upon in vitro restimulation of PBMC collected at month 9; production of IgG against an unrelated antigen (control) is also shown. The dotted line indicates the limit of detection (LOD). Mann–Whitney test was used for assessing statistical differences between groups. ** *p* ≤ 0.01.

SARS-CoV-2 specific B cells were identified among CD19^+/low^ cells by the simultaneous labeling with the spike and RBD probes coupled to different fluorescent dyes (hereafter named S^+^RBD^+^ B cells; Figure 1a; the frequency of RBD-specific B cells among the spike-specific B cells is shown in Supplementary Figure S2). S^+^ RBD^+^ B cells were significantly elicited by vaccination, increasing from 0% at day 0 to 0.15% ± 0.1 of total CD19^+/low^ B cells at day 7 after the second vaccine dose (*p* < 0.001). The frequency of S^+^ RBD^+^ B cells increased over time, reaching values of 0.31% 9 months after vaccination (*p* < 0.001 vs. day 0, Figure 1b). Circulating B cells at month 9 were able to reactivate upon in vitro restimulation and secrete spike-specific IgG, as assessed using memory B cell ELISpot assay. Reactivated memory B cells secreting spike-specific IgG were detected in 82% of the tested subjects, with a mean value of 0.6% of spike-specific secreting IgG with respect to total IgG (Figure 1c). 

### 3.2. Trajectory Analysis of Spike^-^Specific B Cells at Different Time Points

A deep phenotypic longitudinal analysis of total and S^+^ RBD^+^ B cells was performed to define the trajectory of spike-specific B cell response up to 9 months after vaccination. Our flow cytometric analysis was based on a panel that included markers for identifying plasmablasts and different MBCs subsets (CD19, CD20, CD21, CD38, and CD27), as well as the major Ig isotypes (IgD, IgM, IgG, and IgA). A manual gating analysis was firstly performed using CD27, CD21, IgG, IgA, IgM, and IgD markers of all individuals at all time points for defining B cell subsets. These subsets were then combined with a t-Distributed Stochastic Neighbor Embedding (t-SNE) dimensionality reduction algorithm, a tool which groups cells in a bi-dimensional map based on phenotype similarity, thus providing an intuitive and easy approach to view the organization of cell subsets [45,46].

The manual analysis of IgD vs. CD27 expression allows to identify naïve (IgD^+^ CD27^−^) from antigen-experienced B cells which persist as unswitched (IgD^+^CD27^+^) or Ig-switched (IgD^−^CD27^+^) populations, and a double negative subset (DN, IgD^−^CD27^−^). Similar subsets can be identified by analyzing the expression of CD27 vs. CD21. Activated (CD27^+^CD21^−^) and resting (CD27^+^CD21^+^) B cells can be distinguished by naïve (CD27^−^CD21^+^) and atypical (CD27^−^CD21^−^) cells. The surface BCR varied from IgD and IgM double-positive cells to Ig-switched subsets that included IgM (only), IgA, or IgG-positive cells (Supplementary Figure S3).

**Figure 2:**
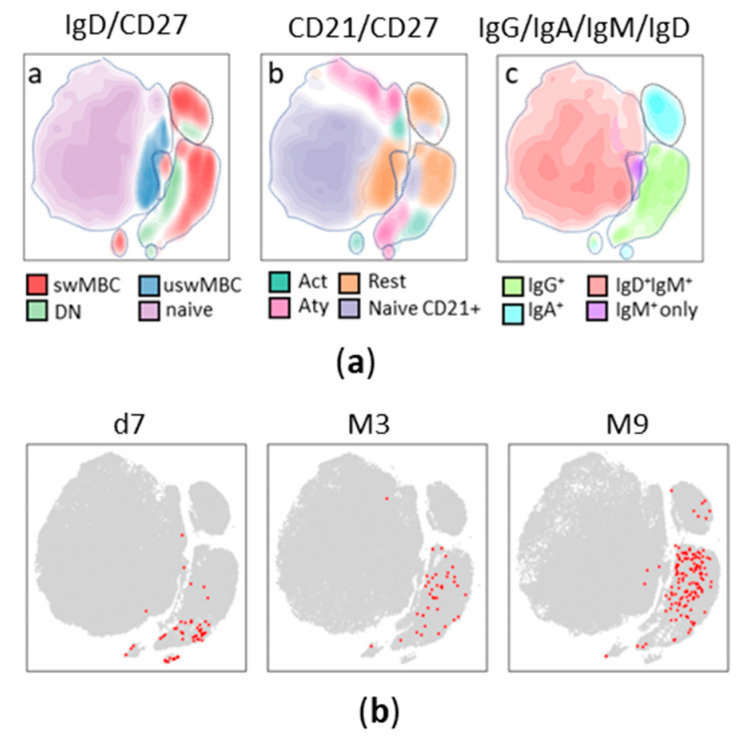
t-SNE analysis of B cell subpopulations and antigen-specific B cells at different time points. (**a**) Different cell subpopulations, according to the expression of different surface molecules, are displayed in the t-SNE map. The left panel displays major B cell subsets according to CD27 and IgD (switched memory, swM; unswitched memory, uswM; double negative, DN; and naïve), middle panel reports B cell subsets according to CD27 and CD21 (activated memory, Act; resting memory, Rest; atypical, Aty; naïve CD21+), right panel reports surface immunoglobulins. (**b**) S^+^RBD^+^ B cells highlighted as red dots in t-SNE dimensionality reduced map, 7 days (d7) and 3 and 9 months after the second vaccine dose (M3 and M9, respectively).

To gain a global picture of the different B cell subsets and compare S^+^RBD^+^ B cells at different time points, we imported the manual analysis into the tSNE map (Figure 2a). Different subsets of total B cells were distributed in different areas of the map. IgD^+^ CD27^−^ naïve B cells occupied a predominant area of the tSNE map, and most of them were CD21^+^ and IgD^+^ IgM^+^ (Figure 2a, panels a, b, and c, respectively). Switched memory B cells were grouped in the right part of the tSNE map (Figure 2a, panel a) and included both IgG^+^ and IgA^+^, with a small fraction of IgM^+^ (Figure 2a, panel c). DN cells included both CD21^+^ and CD21^−^ subsets (Figue 2a, panel b) and were IgG^+^ (Figue 2a, panel c). The S^+^RBD^+^ B cells were scattered in different regions of the tSNE map, according to the three time points analyzed (Figure 2b, red dots). The distribution and the amount of S^+^ RBD^+^ B cells changed over time, suggesting both a quantitative and qualitative modulation of spike-specific B cells at 7 days and 3 and 9 months after vaccine administration. 

**Figure 3:**
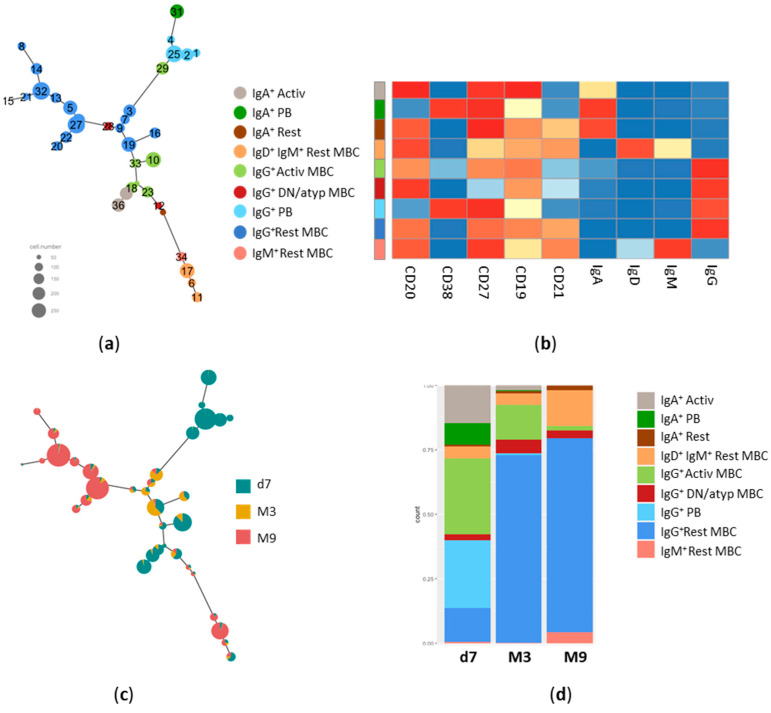
Trajectory analysis of S^+^RBD^+^ B cells. (**a**) Minimum Spanning Tree (MST) of FlowSOM clusters obtained from S^+^RBD^+^ B cells starting from their t-SNE coordinates using CytoTree. Clusters are colored according to their membership in the 9 meta clusters. Size of the nodes is relative to the percentage of cells present in each cluster, as reported on the left. (**b**) Heatmap of markers’ expression within the nine meta clusters, ranging from lower (blue) to higher expression (red). (**c**) Distribution of cells collected at day 7 (d7), 3 (M3), or 9 (M9) months after vaccination within each cluster identified in the MST of panel a. (**d**) Frequency of different phenotypes among S^+^RBD^+^ B cells at day 7 (d7), 3 (M3) and 9 (M9) months after vaccination.

To deeply understand the phenotypes of antigen-specific B cells and their modulation over time, we performed an unsupervised consensus hierarchical clustering analysis implemented into the CytoTree package (Figure 3a). The computational analysis was performed only on S^+^RBD^+^ B cells that were clustered into 36 nodes according to the expression of CD20, CD38, CD27, CD19, CD21, IgG, IgA, IgM, and IgD markers. Phenotypically similar nodes were grouped into 9 meta clusters, colored with the same background, as shown in Figure 3a. The specific expression of each marker within the meta clusters is shown in the heatmap in Figure 3b. Meta clusters included IgA^+^ or IgG ^+^ plasmablasts (PB), activated or resting MBC, IgM^+^-only resting MBC, IgG^+^ DN/atypical B cells, and IgD^+^IgM^+^ unswitched resting MBC. 

Cells of the different time points fell into the different nodes, as shown in Figure 3c. This visualization of the tree clearly highlights the trajectory of the spike-specific B cell phenotypes across the different time points. While IgA^+^ and IgG^+^ PB (nodes 1, 2, 4, 31, and 29 of Figure 3a) and IgA^+^ activated cells (nodes 36 and 18) were present at day 7 after vaccine administration and then disappeared at month 3 and 9, subsets of IgG^+^ activated MBC (nodes 23, 33, and 10) and some clusters of IgG^+^ resting MBC (nodes 3, 7, 9, 19, 16) were detectable both at day 7 and persisted at month 3, while other clusters of IgG^+^ resting MBC (8, 14, 15, 21, 32, 13, 5, 27, 22,20) were strongly detectable only 9 months after vaccination (Figure 3b). Some subsets expressing IgM only (node 34), unswitched-Ig (nodes 17, 6, and 11), or DN/atypical B cells (node 28) were detectable from day 7 and persisted up to month 9. The longitudinal analysis clearly shows a trajectory of the different meta clusters over time, with many of them exhibiting alternative expressions between the early time point (day 7) and the long-term time points (M9; Figure 3b). The relative frequency of the spike-specific B cell within each meta cluster at day 7 and months 3 and 9 is shown in Figure 3d.

In summary, two doses of mRNA vaccine promote an S^+^RBD^+^-specific B cell response that evolves over time, persisting in blood 9 months after vaccination. Spike-specific PB and both IgA^+^ and IgG^+^ were detectable only at day 7, while IgA^+^ and IgG^+^ activated cells were rapidly elicited, downmodulated at month 3, and almost undetectable at month 9. Among persistent RBD-specific B cells, IgG^+^ resting MBC were the predominant phenotype with a small fraction of IgM^+^ IgD^+^ unswitched and IgM^+^-only cells.

### 3.3. Analysis of the Spike^-^Specific IgG Response

**Figure 4:**
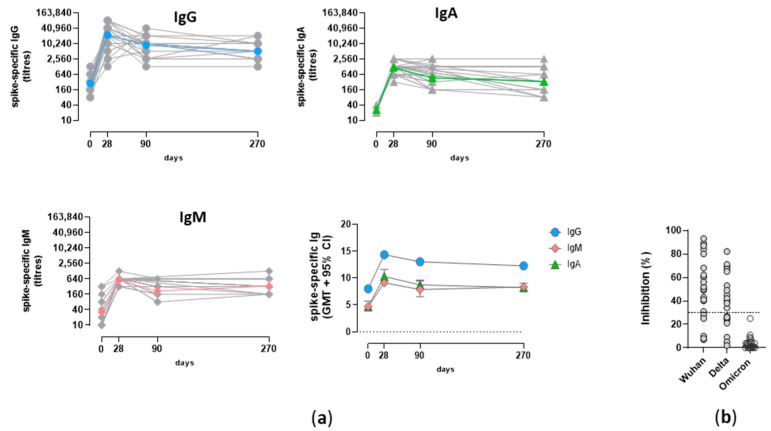
Spike-specific antibody response following SARS-CoV-2 mRNA vaccination. (**a**) Longitudinal analysis of spike-specific IgG, IgA, and IgM response in single subjects detected at baseline (0), 7 days (d7) and 3 (M3), and 9 (M9) months after the second vaccine dose. The GMT value is colored in each graph. In the lower right panel, the GMT of the three antibody classes is shown. (**b**) Surrogate virus neutralization test performed at month 9 against the Wuhan, Delta, and Omicron BA.1 variants. Data are reported as ACE2/RBD binding inhibition percentage. The threshold (dotted black line) at 30% inhibition percentage discriminates between positive and negative samples.

Concomitant to the development of the S^+^RBD^+^ B cell response, the induction and persistence of humoral response was assessed. Spike-specific IgM, IgA, and IgG were longitudinally analyzed (Figure 4a). As expected, the spike-specific humoral response peaked at day 7, declined at month 3, and then stably persisted at month 9. Spike-specific IgG was predominant in each subject, with a peak of geometric mean titre (GMT) 22,185 (95% CI 19,283 to 31,619; range 1280–163,840; *p* ≤ 0.001 versus baseline) after vaccine administration and a value of 5120 (95% CI 2951 to 6280; range 1280–20,480; *p*≤ 0.001 versus baseline) at month 9 (Figure 4a). A very similar trend, but with lower titers, was observed for IgM and IgA antibody response, with a peak of 564 and 1177 GMT at day 7, respectively, and a GMT value of 332 at month 9 (Figure 4a). The induction of IgA and IgG were in line with the detection of IgA^+^ and IgG^+^ PB observed in Figure 3, while the lack of IgM^+^ PB can be due to a very rapid production of IgM^+^ short-lived plasma cells 7–14 days after the first vaccine dose, with a rapid decline. Unfortunately, a cut-off value of circulating antibodies correlating with protection has not yet been identified, also due to the continuous mutation of the virus and the capacity of variants to partially escape the antibody response elicited by vaccination. What we observed in terms of antibody functionality was that in 80% of subjects, the antibodies were able to inhibit the binding between wild-type RBD and ACE-2 receptor, while 54% of subjects had antibodies capable of binding the RBD of the Delta variant and no subject presented antibodies capable of binding the Omicron RBD antigen (Figure 4b). 

## 4. Discussion

In this study, we profiled the spike-specific B cell response after two doses of the mRNA BNT162b2 vaccine in healthy individuals who had no documented history of infection. With a significant proportion of the global population still receiving only two doses of vaccination, there is an urgent need to investigate the durability of the memory response and its cross-reactivity with circulating viral variants. Studying the immune response to COVID-19 vaccines in a real-world setting is complicated by the overlap of recall responses from natural infection with new circulating variants, resulting in what is known as hybrid immunity. In this context, our study cohort represents a valuable group of healthy individuals whose SARS-CoV-2 specific immune response to vaccination was not affected by the viral infection, as evidenced by the absence of antibodies against the viral nucleocapsid. 

The immune response to vaccination typically involves the induction of antibody-secreting cells and serum immunoglobulins, as well as the generation of memory cells that can persist in the host for extended periods [47]. However, vaccination against SARS-CoV-2 has presented unique challenges due to the acute phase of the pandemic, mass vaccination efforts, and the use of new RNA-based vaccine technologies. The first objective of the anti-SARS-CoV-2 vaccine administration was the induction of an effector antibody response capable of neutralizing the virus in the early stages of infection and containing its diffusion, with most studies relying on circulating antibody levels and neutralization activity [48,49]. These data have been particularly important, also considering the adoption, for the first time, of the novel RNA-based vaccine technology; nevertheless, the fundamental role of immunological memory and the importance of investigating and characterizing the B and T cellular responses is now well-recognized. The duration of the memory response is a critical point that can vary depending on the vaccine or antigen. While previous reports analyzed the persistence of the spike-specific cellular response at 6 months post-immunization [21,24,31], here, we profiled the spike-specific B cells trajectory from the initial effector phase (7 days after vaccination) up to 9 months in the absence of natural infection. This is a particularly important point for studying the B cell immune response elicited by the primary cycle of mRNA vaccination without the confounding effects of hybrid immunity elicited by natural infection with SARS-CoV-2 or the impact of a booster dose [25,50,51]. 

Multiparametric flow cytometry is highly effective in conducting in-depth analyses of immune responses following vaccination, as it enables measurement of the frequency, phenotype, and functional characteristics of antigen-specific cells [46]. To identify the different cellular phenotypes, we integrated manual analysis of flow cytometry data with advanced automated tools [45]. S^+^ RBD^+^ B cells were clearly detected in blood 7 days after the second vaccine administration, and they continued to expand over time, after a slow but not significant decline observed at month 3. We can speculate that there is a decrease in circulating antigen-specific B cells immediately after the effector phase, reaching values observed at month 3. However, thereafter, the frequency of spike-specific B cells increases, as reported in other studies [52], possibly due to the persistence of antigen-specific germinal centers observed in the draining lymph nodes [36,53].

This trend can be appreciated in the t-SNE analysis of the spike-specific B cells in the context of the total CD19^+/low^ B cells performed at baseline, 7 days, and 3 and 9 months after the second dose. It can be clearly observed that not only the frequency of S^+^ RBD^+^B cells increased over time, as also reported in other studies [23,31], but that their phenotype changed accordingly. The trajectory analysis of S^+^RBD^+^ B subsets highlighted a clear modulation of specific phenotypes over time, with most of the meta clusters alternatively expressed at day 7 or month 9. The IgA^+^ and IgG^+^ plasmablasts were detected only immediately after vaccination, along with a pool of CD21^−^CD27^+^ IgA^+^ and IgG^+^ activated B cells and a small fraction of IgG^+^ resting memory B cells. However, this scenario transformed over the subsequent weeks, with a reduction in activated B cells and an increase in the resting memory phenotype, positive for IgA or IgG. This is likely due to the transient downregulation of CD21 expression after vaccine administration associated with activated phenotype and its return to higher levels in the subsequent weeks, as recently demonstrated after influenza vaccination [54]. Resting memory B cells became the predominant subset at month 9, with a clear majority of IgG^+^ switched cells, and a small fraction of unswitched (IgM^+^ and IgD^+^) B cells, as well. 

DN/atypical IgG^+^ CD21^−^CD27^−^ B cells were a small subset of spike-specific cells. The DN population has been described as a dominant phenotype in many autoimmune diseases [55], chronic infections such as HIV and malaria [56,57], and in the elderly [58], showing signs of exhaustion and dysfunction. Further studies, however, have demonstrated that they represent a population planned to develop into plasmablasts and that even though CD27^−^ DN cells show signatures of antigen-experienced B cells, such as somatic hypermutation of their Ig genes [59], recently, they have been associated with an alternative lineage primed by primary vaccination and recalled by booster immunization [60]. As observed here, their expansion starts after vaccine administration and then declines at month 9. IgG^+^ MBC circulating at month 9 were able to reactivate and secrete spike-specific IgG in most of the subjects. 

Because the present study is a longitudinal analysis of the spike-specific B-cell response over time, the analysis was performed on frozen/thawed cell samples. This procedure can result in partial damage to cell viability, particularly of the more fragile subtypes such as plasmablasts, thereby reducing the frequency of detectable antigen-specific cells. Nonetheless, the inclusion of CD19^low^ cells in the parent gate is an important strategy for detecting all the plasmablasts that already have downregulated CD19 expression. 

Profiling the induction and persistence of spike-specific MBC in healthy subjects is of primary importance to allow for comparison with the response observed in fragile subjects characterized by an impaired immune system due to concomitant pathologies or immune aging [10,13,61,62]. Studies performed by our group in cohorts of fragile subjects showed that the behavior of the B cell response was different from that of healthy people. In myelofibrosis subjects and individuals transplanted with hematopoietic cells, there was a lower and delayed B cell response [14,15], while people living with HIV generated a rate of spike-specific B cells comparable with healthy controls, but significantly different in phenotype, with a predominant double negative (CD27^−^ IgD^−^) profile [37]. Therefore, the different immune responsiveness to the same vaccine formulation among different cohorts of subjects raises the necessity to carefully consider the vaccination schedules, including the necessity of booster doses, specifically tailored for the different categories of subjects.

In our study, we observed that spike-specific antibodies are still present 9 months after the first vaccination cycle, even though a physiological reduction of the median antibody titer with respect to the peak, measured 7 days after the second dose administration, was detected. As already observed in other studies [63], the stronger drop in antibody response occurred in the first 2 months after the administration of the second dose (here observed between the time points d7 and month 3), but thereafter, it remained at a relatively steady level up to 9 months in most vaccinated subjects [64]. Even with differences in antibody levels, this trend was observed for IgG, IgA, and IgM, in line with other studies [65]. The maintenance of circulating antibodies, especially IgG, 9 months after antigen stimulation can be due to antigen-specific long-lived plasma cells generated within germinal centers upon vaccination with mRNA vaccines [36] and residing in the bone marrow. Concerning the antibody capacity of binding the spike protein and blocking its interaction with the ACE-2 receptor, we observed the binding of the wild-type protein in 80% of subjects, of the Delta variant in 54% (B.1.617.2), of Omicron in none of the subjects (B.1.1.529). Even though the frequency of Omicron-specific B cells was not assessed in this work, other studies have shown the induction of memory B cells cross-reactive against the Omicron variant after 2-dose vaccination [66], with a frequency of about 10% of the bulk spike-specific B cells [67]; this could indirectly explain why the third dose or breakthrough infection significantly boosts the response to the Omicron variant, as reported in other works [66,68,69,70] (Pastore et al. in preparation). 

This study contributes to the characterization of the temporal dynamics and magnitude of the spike-specific B cell response in healthy subjects following the administration of the second dose of the BNT162b2 mRNA vaccine over a 9-month period. When comparing the spike-specific immune responses elicited by mRNA based-vaccines with other COVID-19 licensed vaccine formulations, such as Adenovirus-vectored vaccines (Ad26.COV2.S or ChAdOx1-S) and the recombinant spike protein vaccine (NVX-CoV2373) [31,71], mRNA vaccines were consistently the most immunogenic, both in terms of spike-specific IgG and B cells. Regarding the frequency of RBD-specific memory B cell response, the hierarchy observed was mRNA > Adenoviruses-based vaccines > NVXCoV2373, and significant phenotypical differences were observed, such as the induction of a subset of CXCR3+ memory B cells after Ad26.COV2.S immunization, suggesting a specific functional role in viral vector B cell responses [31]. The deep investigation of the immunological response elicited by novel vaccine platforms performed during the COVID-19 vaccination campaign has provided crucial insights that can aid in the implementation of effective prevention and control measures against SARS-CoV-2.

## 5. Conclusions

Profiling the B cell response is of primary importance for designing and refining vaccination schedules and policies tailored for healthy and fragile subjects. The results of this research provide an important answer to the open question on the duration of the spike-specific memory response upon vaccination with two doses of the novel mRNA-based BNT162b2 vaccine in healthy subjects and provide a clear vision of the trajectory of antigen-specific B cell phenotypes. The future of RNA vaccines is very promising, and novel vaccines against other pathogens are in development; therefore, further data on vaccine immunogenicity are of great relevance.

## Data Availability

The data presented in this study are available in this article (including in the Supplementary Materials).

## Supplementary Materials

The following supporting information can be downloaded at: https://www.mdpi.com/article/10.3390/cells12131706/s1. Figure S1: Nucleoprotein-specific IgG; Figure S2: Frequency of Spike specific B cells recognizing RBD. Figure S3: Manual analysis of total B cell subsets.
