# Supplementary material for: Trajectory of Spike-Specific B Cells Elicited by Two Doses of BNT162b2 mRNA Vaccine
Source: Cells. 2023 Jun 23;12(13):1706. doi: 10.3390/cells12131706 (PMC10340653; doi:10.3390/cells12131706)
Supplement: Supplementary file 1 [file cells-12-01706-s001.zip › cells-2421791-supplementary.pdf]

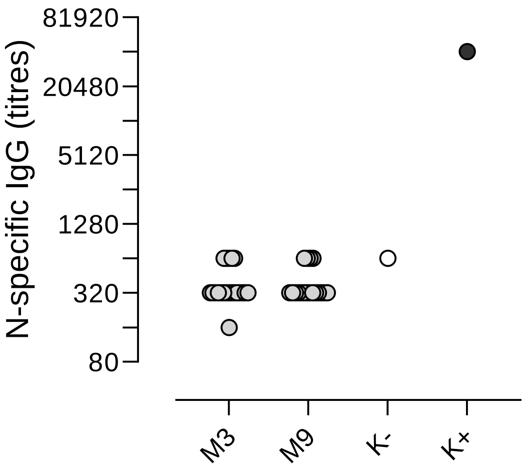

Figure S1. Nucleoprotein-specific IgG. IgG anti-nucleoprotein (N) titers monitored in samples collected at month 3 (M3) and 9 (M9) in each participant to the study. A negative (K-) and positive (K+) controls (WHO International Reference Panel of anti-SARS-CoV-2 immunoglobulin, NIBSC code 20/144 and 20/142 respectively) were included as reference. Antibody titres are expressed as the reciprocal of the dilution of sample reporting a double OD value compared to the background.

A

A

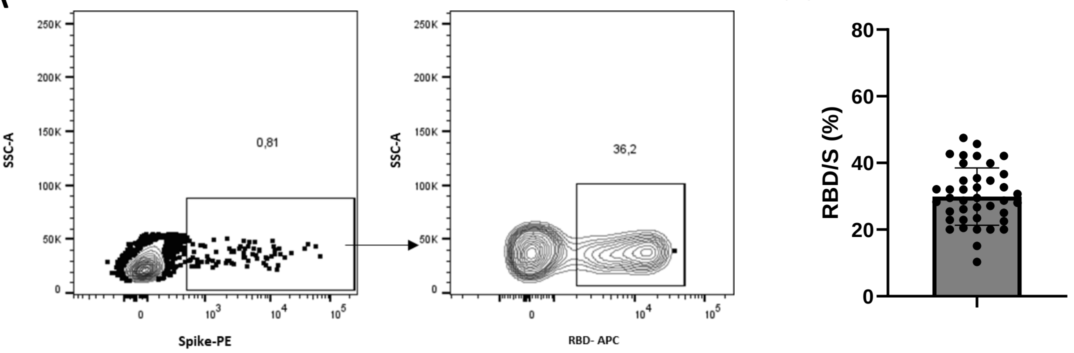

Figure S2. Frequency of Spike-specific B cells recognizing RBD. A) Flow cytometry analysis of live CD19$^{+/low}$ B cells, positive for spike antigen, and then for RBD region. B) Frequency of RBD-specific B cells respect to spike-specific B cells in all samples.

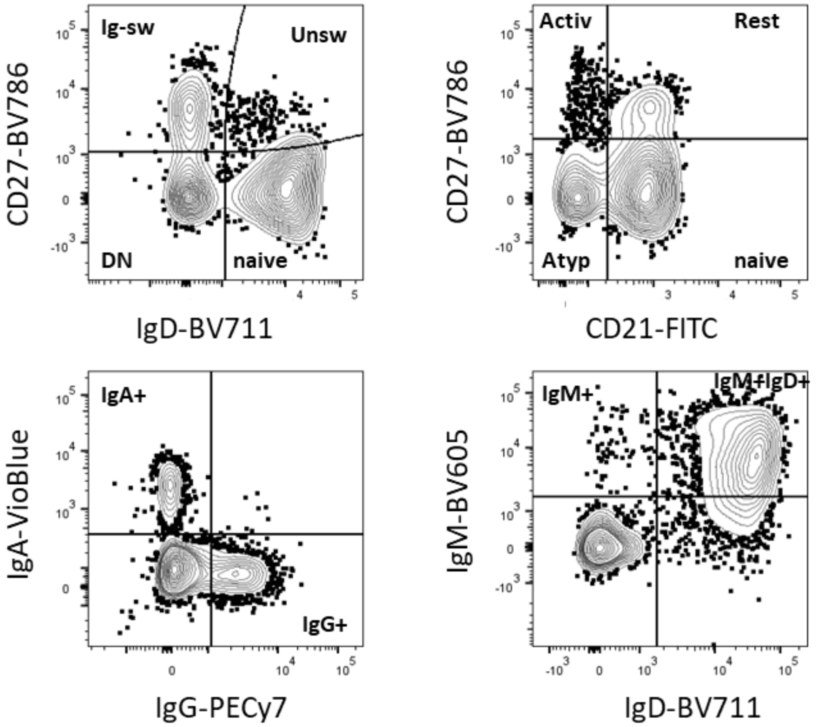

Figure S3. Manual analysis of total B cells subsets. Flow cytometry analysis of live CD19$^{+/low}$ B cells, according to the expression of IgD *versus* CD27 (CD27^{+}IgD^{-} switched memory, Ig-sw; CD27^{+}IgD^{+} unswitched, Unsw; CD27^{-}IgD^{-} double negative, DN; and CD27^{-}IgD^{+} *naïve*), CD21 *versus* CD27 (CD27^{+}CD21^{-} activated, Activ; CD27^{+}CD21^{+} resting, Rest; CD27^{-}CD21^{-} atypical, Atyp; and CD27^{-}CD21^{+} naïve) and IgG^{+}, IgA^{+}, IgD^{+}IgM^{+}, IgM^{+} only.
